# Precision Nutrition Model Predicts Glucose Control of Overweight Females Following the Consumption of Potatoes High in Resistant Starch

**DOI:** 10.3390/nu14020268

**Published:** 2022-01-09

**Authors:** Joy V. Nolte Fong, Derek Miketinas, Linda W. Moore, Duc T. Nguyen, Edward A. Graviss, Nadim Ajami, Mindy A. Patterson

**Affiliations:** 1 Department of Nutrition Sciences, Texas Woman’s University, Houston, TX 77030, USA; DMiketinas@twu.edu (D.M.); mpatterson14@twu.edu (M.A.P.); 2 Department of Surgery, Houston Methodist Hospital, Houston, TX 77030, USA; lwmoore@houstonmethodist.org (L.W.M.); eagraviss@houstonmethodist.org (E.A.G.); 3 Department of Pathology and Genomic Medicine, Houston Methodist Research Institute, Houston, TX 77030, USA; dtnguyen6@houstonmethodist.org; 4 Department of Genomic Medicine, University of Texas MD Anderson Cancer Center, Houston, TX 77030, USA; NAjami@mdanderson.org; 5 Institute for Women’s Health, Texas Woman’s University, Houston, TX 77030, USA

**Keywords:** resistant starch, potatoes, microbiome, *Faecalibacterium*, precision nutrition

## Abstract

Individual glycemic responses following dietary intake result from complex physiological processes, and can be influenced by physical properties of foods, such as increased resistant starch (RS) from starch retrogradation. Predictive equations are needed to provide personalized dietary recommendations to reduce chronic disease development. Therefore, a precision nutrition model predicting the postprandial glucose response (PPGR) in overweight women following the consumption of potatoes was formulated. Thirty overweight women participated in this randomized crossover trial. Participants consumed 250 g of hot (9.2 g RS) or cold (13.7 g RS) potatoes on two separate occasions. Baseline characteristics included demographics, 10-day dietary records, body composition, and the relative abundance (RA) and α-diversity of gut microbiota. Elastic net regression using 5-fold cross-validation predicted PPGR after potato intake. Most participants (70%) had a favorable PPGR to the cold potato. The model explained 32.2% of the variance in PPGR with the equation: 547.65 × (0 [if cold, high-RS potato], ×1, if hot, low-RS potato]) + (BMI [kg/m^2^] × 40.66)—(insoluble fiber [g] × 49.35) + (*Bacteroides* [RA] × 8.69)—(*Faecalibacterium* [RA] × 73.49)—(*Parabacteroides* [RA] × 42.08) + (α-diversity × 110.87) + 292.52. This model improves the understanding of baseline characteristics that explain interpersonal variation in PPGR following potato intake and offers a tool to optimize dietary recommendations for a commonly consumed food.

## 1. Introduction

Resistant starch (RS) is a bioactive fiber found naturally in certain foods. Health benefits associated with RS intake include lower postprandial glucose and insulin and an improvement in enteroendocrine hormones [1,2,3,4,5,6,7,8,9]. Potatoes contain RS, which can increase in concentration based on preparation method [10]. Cooked then chilled potatoes contain higher amounts of RS compared to cooked, nonchilled potatoes, through the process of retrogradation [11]. The process of altering preparation methods to optimize RS from potatoes, one of the most commonly consumed high-RS foods [12], had not been investigated to promote a glycemic benefit until recently [13]. Previous studies demonstrate an improvement in glycemic biomarkers following the intake of Russet potatoes with varying RS concentrations that resulted from different cooking and preparation methods. Significant reductions in insulin and glucose-dependent insulinotropic peptide (GIP) area under the curves (AUC) 120 min postprandial were found following the cold potato compared to a hot potato; however, not all of the participants responded favorably to the cold potato in terms of lower postprandial glucose response (PPGR).

Foods with higher amounts of RS, or fiber, typically have lower glycemic index (GI) scores compared to those with more rapidly digestible starches. Gaesser et al. investigated the impact of fiber on postprandial glucose AUC in healthy adults, and found that low GI meals resulted in 9% lower 4 h glucose AUC [14]. Other studies have shown that foods with higher levels of RS through gelatinization (i.e., cooling after cooking) have a lower glycemic index, or reduced available carbohydrate, which may help explain why Patterson et al. observed a lower glucose iAUC following the cold potato compared to the hot [15]. Consuming foods with a lower GI may help improve glucose homeostasis and reduce risk factors for metabolic syndrome [16,17], type 2 diabetes [18] and cardiovascular disease [19,20]. However, controversy remains regarding the impact of a low-GI diet or improved PPGR. For example, the OmniCarb randomized control trial did not indicate improved cardiovascular markers after consuming a low-GI diet for 5 weeks [21].

An area of interest in better understanding the influence of RS on postprandial glucose homeostasis is its relationship with the microbiome. Key microbiota from the *Ruminococcus* and *Eubacterium* genera and *Faecalibacterium prausnitzii* species degrade RS, which promotes the production of short-chain fatty acids (SCFAs) [22,23]. Several reviews describe how RS modifies the microbiome; however, an important distinction should be made for the reverse scenario: how the microbiome environment influences the physiological responses to RS. To consider the influence of the microbiome on glycemic response to foods, in particular to RS, focus has been placed on SCFA binding to receptors of enterocytes to stimulate incretins, such as GIP and glucagon-like peptide-1 (GLP-1) [24,25,26]. Downstream reactions release GLP-1 and in turn stimulate insulin secretion. Other studies indicate that select microbiota can predict PPGR independently of other host metabolic and/or physiologic factors [27], and that select dietary fibers (i.e., RS4) induce divergent and highly specific modulations of the microbiome to alter the output of propionate or butyrate SCFAs. Thus, the microbiome can modulate the postprandial response from consuming RS. 

To better understand interindividual differences, the field of precision nutrition identifies factors that contribute to individual responses to dietary interventions. Most studies investigating responses to RS interpret results as collective means, and do not consider variability among individuals or factors driving interindividual differences. Since postprandial physiological responses depend on several complex biological and metabolic processes, this oversimplification of PPGR warrants deeper investigation with consideration for the individuals’ specific biology and external influences (i.e., dietary choices). A precision nutrition framework takes interindividual differences into account using machine learning, and has been used to predict PPGR following dietary interventions. For example, Zeevi et al. [28] investigated PPGR in over 800 participants to develop a predictive model for selecting dietary recommendations to lower PPGR. The model’s prediction of foods to lower glycemic responses in a prospective cohort was accurate and superior to standard dietary recommendations.

PPGR has also been predicted following the consumption of specific foods. Korem et al. [27] conducted a crossover intervention in healthy subjects using white and sourdough breads (4 g vs. 9 g of fiber, respectively) and compared PPGR and other clinical parameters. Each bread intervention provided 50 g of available carbohydrates. Data analysis utilizing linear mixed models found no significant differences in clinical parameters (i.e., glucose, insulin, or low-density lipoprotein) between the two groups. However, upon deeper investigation of person-specific responses, not all participants exhibited lower PPGR following the intake of the higher-fiber sourdough bread. A model using gradient boosting regression and leave-one-in cross-validation identified factors that predicted PPGR. Of note, baseline microbiome features, including relative abundance (RA) of species, RA of genes, and function, solely predicted PPGR following the intervention. Thus, integrating models that include person-specific factors may predict individual PPGR better than traditional statistical methods. Key variables used in these personalized models to predict PPGR include demographics, dietary intake, and microbiome composition.

The objectives of the present study included assessing baseline characteristics to predict PPGR in individuals following an intervention of low- vs. high-RS potatoes (hot vs. cold Russet potatoes, respectively) and to develop a precision nutrition model to predict PPGR. To achieve these objectives, we performed a post hoc analysis and used an elastic net penalty to select variables to be included in the model. Baseline characteristics included demographics, dietary components, body composition, the RA of stool microbiota, and α-diversity of the stool microbiome. We hypothesized that our model would identify predictors of PPGR, and that microbiome features would significantly contribute to the explained variance of the model.

## 2. Materials and Methods

### 2.1. RS Quantification

Quantification of RS was performed using the AOAC Method 2002.02 (Megazyme© RS Assay Kit, Bray, County Wicklow, Ireland) [29]. Three potatoes were prepared for each potato cooking method: baked—served hot, boiled—served hot, baked—chilled, and boiled—chilled. Chilled potatoes were stored at 4 °C for 5 days. After potatoes were prepared, they underwent lyophilization for 72 h to ensure adequate drying. Each potato type was run in duplications for 2 samples taken from each potato (a total of 4 per potato type). The mean concentrations of RS per potato type were compared using the unpaired t-test with significance being *p* < 0.05.

### 2.2. Glucose Response

Blood samples were drawn at fasting (>8 h, water allowed) and at 15, 30, 60, and 120 min postprandial. Blood was collected in one 6 mL EDTA vacutainer for glucose. All blood samples were centrifuged at 4000 RPMs for 15 min. Serum was immediately aliquoted into cryovials and stored at −80 °C. Serum glucose was determined by colorimetric analysis. Quantification of glucose occurred by a multimode reader (Synergy HI, BioTek^®^ Instruments, Inc., Winooski, VT, USA).

The incremental area under the curve (iAUC) was calculated for glucose from fasting until 120 min after the final bite of the potato. The area between each time interval (15–0 min, 30–15 min, 60–30 min, and 120–60 min) was calculated using the trapezoidal method based on 5 different equations, depending on if concentrations fell above or below the baseline concentration. Only the area above the baseline value was retained in the iAUC value. Detailed equations and scenarios of iAUC calculations are described elsewhere [30,31]. The iAUC describes the cumulative postprandial response over time; however, important features such as the concentration maximums (Cmax) and minimums (Cmin) between each intervention are not independently captured. Therefore, we tabulated the Cmax and Cmin and the time to Cmax and Cmin (as Tmax and Tmin, respectively) of glucose and compared them between potato interventions.

### 2.3. Clinical Trial and Baseline Characteristics

More detailed descriptions of the study participants and the primary analysis can be found elsewhere [13]. In brief, participants were all females with a body mass index (BMI) between 25 and 40 kg/m^2^. Participants were excluded if they were not between 18 and 40 years old, were pregnant or lactating, had significant weight change in the past 6 months, or were taking medications or supplements that affect metabolism or antibiotics/probiotics within the past 3 months.

Body composition was measured using air displacement plethysmography via the BOD POD^®^ (COSMED USA, Inc., Concord, CA, USA) to determine fat mass and fat-free mass. Anthropometrics included height, weight, and BMI. The weight measurement used was derived from the calibrated scale from the BOD POD^®^.

Dietary records were captured over 10 days throughout the study to better represent a participant’s usual diet compared to other methods such as 24 h recalls. One participant had an average energy intake of >4000 kcals/d and was likely an over-reporter [32]; therefore, we applied a crude cutoff value of 4000 kcals/day and adjusted subsequent dietary components to the proportion of energy reduction. Dietary information was entered into the Nutrition Data System of Research (Nutrition Coordinating Center, University of Minnesota, 2016), and the nutrient composition was analyzed. Only dietary components thought to be relative to the study question were included for analysis.

Participants collected stool specimens prior to the first study intervention (OMNIgene^®^ GUT OM-200, DNA Genotek, Ontario, CA, USA). Stool samples were aliquoted and stored in −80 °C freezer and batch-analyzed by MicrobiomeDx (Houston, TX, USA). In brief, microbial DNA was extracted using the Mag-Bind Universal Pathogen DNA Kit (Omega Bio-Tek, Norcross, GA, USA). 16S sequencing libraries were generated by amplifying the v3–v4 hypervariable regions of the 16S gene [33]. MicrobiomeDX used BacPro™, a proprietary algorithm, to generate a comprehensive report that includes α-diversity scores describing community richness, evenness, taxonomic composition with RA.

### 2.4. Statistical Analysis

No participants or periods were excluded following assessments of the carryover, treatment, and period effects, demonstrating an adequate 7-day washout period. We applied simple mean imputation for missing biomarkers of one participant [34]. Outliers of biomarkers were evaluated visually by boxplots and calculated by interquartile range (IQR) × 3.

Normality assumptions were evaluated using the Shapiro–Wilk test, and hypothesis testing was performed based on the distribution. Demographics (age, ethnicity, BMI) were described using proportions and mean (standard deviation). The glucose iAUC was calculated using the trapezoid method, and differences in biomarkers between the potato interventions were determined by Wilcoxon signed rank test and described as median (interquartile range). Dietary data were calculated as means and standard deviations or medians and interquartile range, as appropriate, to describe the energy, total and percent of kilocalories of macronutrients (fat, protein, and carbohydrates), available carbohydrate, glycemic index, total fiber, insoluble fiber, soluble fiber, monounsaturated fatty acids, polyunsaturated fatty acids saturated fatty acids and trans fatty acids. Microbiota taxa included in correlative studies and the regression model were selected based on prevalence and use in previous literature. At a minimum, genera had to be present in at least 50% of the participants. Five phyla and 1 family of interest were also included based on prior studies related to PPGR. Correlations between glycemic biomarkers and baseline demographics, body composition, microbiota, and diet, were performed using Spearman’s rho. Relationships between the microbiota and biomarkers included a Bonferroni correction for multiple comparisons. All other significance was assigned at *p* < 0.05. Statistical analysis was performed using Stata^®^ v.16.1 (College Station, TX, USA), and figures were generated using Prism v.7.03 (Graphpad, San Diego, CA, USA).

### 2.5. Penalized Regression

We used a data-driven approach to build a penalized regression model with an elastic net penalty and k-fold cross-validation to identify predictors of PPGR following consumption of low-RS (hot) and high-RS (cold) potatoes. Baseline demographics, body composition, Shannon and Simpson α-diversity, the RA of key microbiota, and dietary intake were used as input data for the models (Supplemental Table S1). Predictors that were sparse or clinically insignificant were removed as input variables.

One model was built to predict glucose iAUC with a variable in the final equation to account for potato type (low-RS vs. high-RS). Data was stacked, and the glucose iAUC for low-RS (1) or high-RS (0) potatoes were retained as separate input variables; all other variables were duplicated and controlled for by using the vce cluster option in STATA^®^ (variance-covariance matrix of estimators, used commonly in case–control analyses) for matched-paired comparison. A 5-fold cross-validation was used to avoid overfitting the model. The strength of this approach is the out-of-sample prediction [35]. This method of k-fold cross-validation randomly partitions data into k − 1 samples for training and then tests the model on the 1 held out k-fold. Elastic net regression was chosen over other penalized regression methods because it applies two penalty terms, a combination of the L1 norm from the least absolute shrinkage and selection operator (LASSO) to provide feature selection and the L2 norm of ridge regression to provide effective regularization. This makes it optimal for analysis using a small sample size and a large number of predictors that are highly correlated (i.e., dietary components and microbiota composition). After elastic net variable selection, we performed linear regression with the final input variables to generate β-coefficients and the model equation.

The linearity of the standardized residuals against each of the predictor variables in the regression model was evaluated. Although there is a certain level of nonlinearity at the far end values of BMI, insoluble fiber, and Bacteroides RA, we believe it is not severe, and it is acceptable that the final model met the linearity assumption. The normality of residuals of the final model was evaluated using the Shapiro–Wilk test for normality. With *p* = 0.16, the Shapiro–Wilk test indicated that the residual of the final model was normally distributed. Correlations between dietary components and the microbiome were present; however, the implementation of the elastic net penalties controls for multicollinearity by restricting correlated parameters so that only one (the most predictive) is retained in the model [36]. To this effect, the variance inflation factor (VIF) revealed no evidence of multicollinearity. Lastly, the vce cluster option to fit the model resulted in no homoscedasticity concerns.

## 3. Results

### 3.1. Participants and Study Design

A total of 30 overweight females without comorbid conditions participated in this randomized crossover study. Participants consumed roughly 9.2 ± 1.1 g of RS during the low-RS (hot) potato intervention and 13.7 ± 3.0 g of RS during the high-RS (cold) potato intervention (*p* = 0.009). The mean age of participants was 29.6 ± 6 years old, and the mean BMI was 32.8 ± 3.6 kg/m^2^. Participants exhibited a high body fat percentage, averaging 45.5 ± 4.8%. The following results consist of a post hoc analysis describing baseline characteristics associated with PPGR and the predictive model developed using key baseline features.

### 3.2. Postprandial Biomarker Response

Postprandial responses following the low-RS and high-RS potatoes varied among participants. Previous analysis [13] revealed reductions in glucose concentration following the high-RS potato in the early postprandial period (15 and 30 min) compared to the low-RS potato, but no difference in glucose total area under the curve (tAUC) between potatoes occurred. However, when the data were reanalyzed based on incremental AUC (iAUC) to exclude values below the basal fasting concentrations from the AUC calculation [37], PPGR was different between potato intakes, *p* = 0.02.

Despite an overall significant reduction in the median glucose concentration of the group, not all participants demonstrated a lower glycemic response following the high-RS potato (Table 1). The median reduction in glucose iAUC after consuming the high-RS potato was 471 mg·h/mL, *p* = 0.02. Twenty-one out of 30 participants (70%) exhibited a lower glucose iAUC after consuming the high-RS potato compared to the low-RS potato.

### 3.3. Dietary Patterns

Dietary patterns heavily influence the gut microbial population and epigenetic factors associated with glucose metabolism [38]; therefore, key components of dietary intake contributed to the model. Dietary records were captured over 10 days throughout the study to represent the usual diet of participants during the study period (six weekdays and four weekend days). Participants consumed an average of 1828 ± 643 kcals/d (Table 2). Dietary patterns revealed slightly higher fat and sugar intakes than the U.S. Dietary Guidelines for Americans 2020–2025 recommendations [39]. The mean percent of kilocalories (%kcals) from fat totaled 36% (recommended between 20 and 35%), and the mean saturated fat intake was 25.4 ± 12.7 g/day (recommended as <10 g/day). Further, participants consumed a diet high in sugar, with 48 g out of 75 g (64.1%) of sugar ingested as added sugars (sugar not naturally present in the food product consumed). Added sugars composed 10.5% of kcals consumed per day, which slightly exceeds dietary recommendations of 10% of total kcals. Fiber intake did not meet dietary guidelines and averaged nearly half of the recommended intake: 15.24 g/day consumed by the participants vs. recommendations of 25 g/d [39]. Of note, RS is not a dietary component that is currently assessed in food databases, and therefore, the usual intake of RS could not be reported in this study.

### 3.4. Microbiome Profile

**Figure 1:**
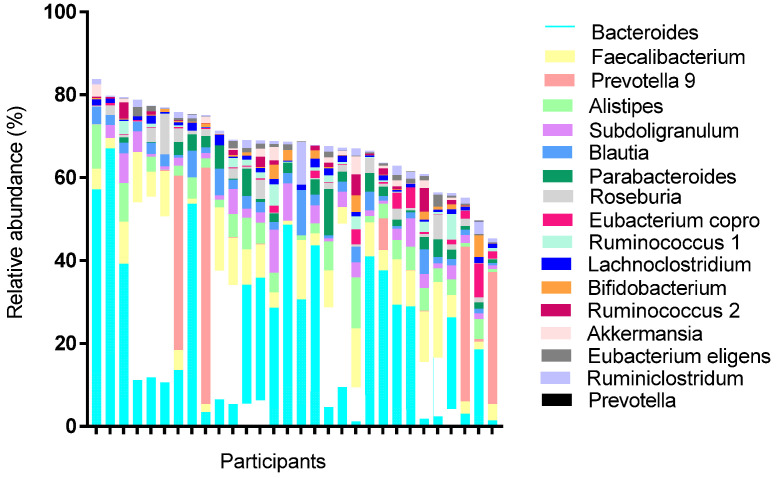
Relative abundance per participant of genera classified as key microbiota.

**Figure 2:**
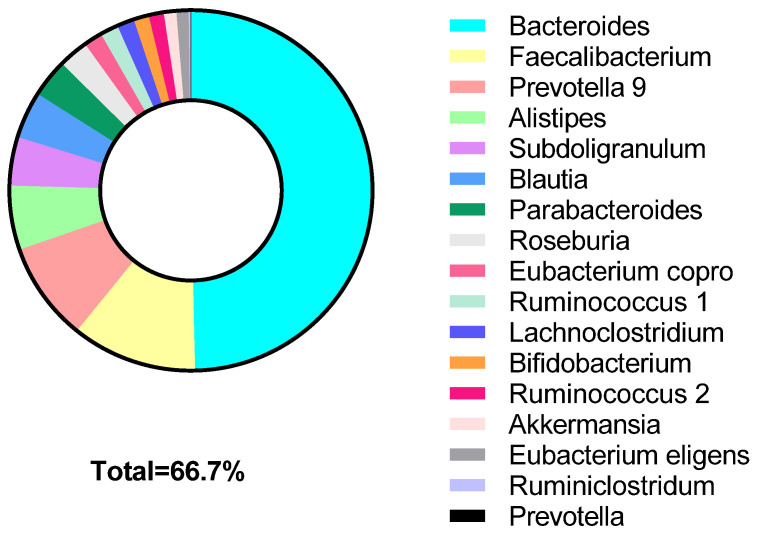
Cumulative relative abundance of genera classified as key microbiota. The selected taxa represent 66.7% of the abundance in participants.

Microbiome profiling using 16S rRNA occurred from a single stool sample collected prior to the first potato intervention. All 30 participants provided an adequate sample for analysis. The average number of operational taxonomy unit reads was 147,070, with 75% of those reads mapped to the SILVA database as previously observed microbes [40]. Divided by taxonomy, there were 14 different phyla, 62 families, and 221 genera identified. Top phyla detected followed typical Western patterns, and included: Firmicutes, Bacteroidetes, Proteobacteria, and Actinobacteria. The most prevalent genera (phyla) observed among the samples were Bacteroides (Bacteroidetes), Faecalibacterium (Firmicutes), Blauti (Firmicutes), Lachnoclsotridium (Firmicutes), Ruminococcus (Firmicutes), Anaerostipies (Firmicutes), and Ruminiclostridium (Firmicutes; Figure 1). Relative abundance of top taxa varied considerably between participants. Key taxa used for correlative and predictive analyses were determined based on previous studies using microbiota to predict PPGR (Supplement Table S1) [28,41]. The genera included in the penalized regression model accounted for >66% of the total population genera (Figure 2).

### 3.5. Correlative Relationships with Baseline Characteristics and Glucose iAUC

All significant relationships between baseline features and PPGR differed for low- and high-RS (Table 3), meaning that the different RS content in the potatoes played a major role in these findings. Following the low-RS potato, moderate, inverse relationships existed between height and the RA of *Faecalibacterium*. These correlations were not found from the high-RS potato intervention. Glucose iAUC following the high-RS potato correlated inversely to insoluble fiber intake and the RA of the Actinobacteria phyla. Positive correlations were seen between %kcals from fat and protein and the high-RS potato glucose iAUC. Of note, when a Bonferroni correction factor was applied to the microbiome relationships with glucose iAUC, no significant correlations remained. These differences in PPGR following each potato, though consumed by the same participants, further highlights the need for precision nutrition to distinguish personal characteristics that influence PPGR.

### 3.6. Predictve Model for PPGR following Potatoes

A total of 58 input variables (Supplemental Table S1) were used for elastic net selection for the final model. Stopping criteria and variable selection were based on 5-fold cross-validation. Sparse taxa were removed. The top five most abundant genera in our sample and taxa used in previous prediction models for postprandial glucose were evaluated [27,28,41,42]. Note that due to the unavailability of RS concentration in food databases, we were not able to include ratios related to RS, such as carbohydrates:RS or insoluble fiber:RS, in the model. Variables selected by elastic net regression were included in the final model, even if they did not yield significant findings, namely because of their clinical significance and contribution in explaining the model’s variance. The root-mean-square error, or the prediction error that was minimized by the model hyperparameters, was 855.32.

Glucose iAUC could be explained by the potato type (low-RS vs. high-RS), three genera, Simpson α-diversity, BMI, and insoluble fiber intake. The model accounted for 32.2% of the variance (R^2^) in glucose iAUC. Only the type of potato (low-RS or high-RS) and the RA of *Faecalibacterium* were significantly associated with glucose iAUC (β = 547.65, 95% CI 131.61, 963.68, *p* = 0.01 and β = −73.49, 95% CI −128.51, −18.47, *p* = 0.01, respectively; Table 4).

The type of potato (low-RS vs. high-RS) was the only significant, positive association with glucose iAUC, while the RA of *Faecalibacterium* significantly and negatively associated with glucose iAUC. This model predicts the PPGR following either low- or high-RS Russet potatoes in this small population of overweight women.

## 4. Discussion

This study observed significant reductions in glucose iAUCs following the intake of high-RS potatoes compared to low-RS potatoes in 30 overweight or obese women. This study is novel in that a predictive equation to determine baseline characteristics that influenced the glycemic response following a low- or high-RS potatoes was developed. The study focused on understanding the glycemic benefits of modifying RS by cooking and refrigeration in a commonly consumed food.

Investigations into the role of RS on glucose homeostasis related to other RS foods mirror the results of the current study. A randomized crossover trial by Nilsson et al. [3] examined a 3-day intervention where high RS bread (barley, ~17 g RS/day) was compared to white bread (2.5 g RS/day) and reported improved glucose homeostasis in 20 healthy volunteers (85% women). Postprandial glucose peaks were reduced following the high-RS bread. Stewart et al. [43] provided acute supplementation of RS type 4 (16.5 g) in a crossover study comparing high fiber vs. low fiber scones. After measuring the iAUC for 180 min, postprandial glucose significantly reduced between 43 and 45% [43]. However, these trials did not examine the influence of food processing on RS levels in whole foods, nor was the influence of the gut microbiome explored. These trials, among many others [1,2,6,7,43], demonstrate that a higher, acute intake of RS resulted in improved PPGR. It is important to note that the present study observed reductions in glucose iAUC following the intake of a high-RS potato with approximately 13.7 g of RS, which is a lower amount than many of the studies, but was still efficacious. This in part may be due to the combination of RS types, both RS type 2 and RS type 3, present in the high-RS potato, or other fiber components inherent in potatoes. We also studied healthy, overweight females following acute ingestion of a whole food product containing RS, rather than a RS supplement, which few other studies exclusively investigate.

The usual dietary intake reported by the study participants mostly aligned with the U.S. Dietary Guidelines for Americans 2020–2025 [39]. The most concerning dietary pattern from our participants was the lack of fiber intake and excessive added sugar consumption. Studies investigating the postprandial response of RS (either acute or prolonged consumption) usually fail to report the usual dietary intake of the participants in the trial. Our participants’ metabolic phenotype was primed by diets high in available carbohydrate, possibly confounding the interaction between epigenetic factors and the low-RS potato (higher in available carbohydrates). Phenotypic patterns may have affected the availability of digestive enzymes or activated genes related to carbohydrate metabolism or RS degradation. RS causes genetic alterations in carbohydrate metabolism [44], to where participants consuming a higher-fiber diet may have elicited a different response to the intervention than individuals with consistently lower-fiber intake. The importance of this may be evident in the negative association (though not significant, *p* = 0.14) between insoluble fiber and glucose iAUC in our regression model.

The interplay between diet and gut microbiota may play an important role in how dietary RS can improve PPGR. Although this study did not measure the fermentation of RS by gut microbiota nor the microbiome changes resulting from RS intake, we did measure the association between baseline microbiota and PPGR. Several genera showed relationships with postprandial glucose iAUC following consumption of low- and high-RS potatoes. The *Faecalibacterium* genus and Actinobacteria phyla showed moderate, negative correlations with glucose iAUC following the intake of low- and high-RS potatoes, respectively. The results of this observational trial should be tested in greater depth in randomized controlled trials and mechanistic studies.

Several studies have reported similar relationships between *Faecalibacterium* and glucose. Zhang et al. sequenced the microbiome of patients with different levels of glucose intolerance, and *Faecalibacteria prausnitzii* was most abundant in the normal glucose-tolerant group compared to the participants with prediabetes and type 2 diabetes mellitus (T2DM) [45]. The influence of *Faecalibacterium* on glucose iAUC became evident in our model as the only significant contributor, other than potato type, associated with PPGR. Other studies have also observed an inverse relationship between *Faecalibacterium* and diabetes [46]. A recent review [47] reports that four out of five studies found a negative association between *Faecalibacterium* and T2DM. To our knowledge, this is the first study to demonstrate the inverse association between *Faecalibacterium* and iAUC glucose following a high-RS whole food.

Various modeling techniques can predict PPGR using baseline characteristics. In a post hoc analysis of 106 healthy Danish adults, Sᴓndertoft et al. used a random forest model focused on clinical features and the microbiome to determine the effect on PPGR [42]. The authors noted that a model based solely on microbial components accounted for up to 14% of the variance in PPGR excursions. When clinical features were added to the model, up to 78% of the variance in PPGR excursions was reported. Our model did not have any other clinical laboratory values available, such as serum cholesterol or triglycerides, which may have explained more variance. Because many underlying clinical, physiological, and metabolic features contribute to PPGR, stronger predictors may exist that were not assessed in the present study.

In the present study, a key element in the design was to provide a dietary intervention that could feasibly be achieved in a real-world setting. The amount of potato administered (250 g) was equivalent to a serving size of mashed or baked potato, which can be realistically consumed alone or with a meal. We were unable to determine the actual amount of RS2 and RS3 (RS3 exclusively in the cold potato) for each potato administered to participants, but we did quantify the mean RS in the hot and cold potatoes. Moreover, the volume of potato consumed was equivalent between interventions, yet the proportion of available carbohydrate differed, with a lower amount of available carbohydrate in the cold, high-RS potato. Another limitation of this study includes that the postprandial time period did not allow for adequate assessment of bacterial fermentation of RS and further stimulation of incretins located lower in the gut. We were also limited to microbial data at the 16S rRNA level, while whole-genome sequencing could provide deeper insight into the functional role of key microbiota and specific species associated with lowering postprandial glucose. Despite these limitations, a robust modeling technique incorporated common baseline features and selected variables with the greatest influence on PPGR. Further, because this study recruited volunteers without chronic disease, the identified predictors of PPGR may be applicable to other healthy populations.

## 5. Conclusions

Incorporating simple, modifiable changes, such as increasing the RS concentration in the commonly consumed potato by changing the cooking method, may aid in better control of glycemic responses to this starchy food. Understanding the interpersonal variation in the glycemic effect of potatoes would allow for appropriate dietary recommendations and optimization of routine food choices. The gut microbiota, especially *Faecalibacterium*, predicted the PPGR following potato intake. Larger studies are needed for the generalizability and evaluation of more diverse populations.

## Figures and Tables

**Table 1:** Postprandial glucose response of women with overweight (*n* = 30) following hot and cold potato consumption.

Postprandial Glucose	Low-RS Potato	High-RS Potato	Delta (Low − High)	*p*-Value
iAUC, mg·h/mL	1180 (500, 1910)	709 (316, 1038)	471	0.021
Concentration maximum, mg/dL	153.2 (129.6, 174.7)	140.93 (124.6, 160.8)	12.3	0.047
Concentration minimum, mg/dL	95.93 (85.2, 120.9)	100.70 (89, 109)	−4.8	0.417
Time to peak concentration, minutes	30 (15, 30)	30 (15, 30)	0	0.767
Time to minimum concentration, minutes	120 (22, 120)	90 (30, 120)	30	0.99

All values are presented as median (interquartile range) except for the delta. iAUC, incremental area under the curve; RS, resistant starch.

**Table 2:** Mean 10-day nutrient composition.

Dietary Variable	Mean (SD)
Energy, kcal	1828 (643)
Total fat, g	78.7 (36.1)
Kcals from fat, %	36.0 (5.1)
MUFA, g	27.9 (12.4)
PUFA, g	18.8 (9.1)
Trans FA, g	2.3 (1.17)
SFA, g	25.4 (12.7)
Protein, g	75.0 (28.0)
Kcals from protein, %	16.6 (3.5)
Total CHO, g	206.4 (69.2)
Kcals from CHO, %	45.5 (8.1)
Total sugar, g	75.4 (36.9)
Added sugar, g	48.1 (27.8)
Available CHO, g	191.1 (66.7)
Total Fiber, g	15.2 (5.7)
Soluble fiber, g	4.5 (1.5)
Insoluble fiber, g	10.7 (4.4)
Glycemic Index	60.3 (4.6)

Dietary record information was inputted into the Nutrition Data System for Research (Nutrition Coordinating Center, University of Minnesota, 2016) to determine nutrient composition. Abbreviations: CHO, carbohydrate; kcal, kilocalories; MUFA, monounsaturated fatty acids; PUFA, polyunsaturated fatty acids; SFA, saturated fatty acids.

**Table 3:** Significant * correlations between baseline characteristics and iAUCs of glucose, Spearman’s rho.

	Glucose–Low-RS Potato		Glucose–High-RS Potato	
	Rho	*p*-Value	Rho	*p*-Value
ANTHROPOMETRICS				
Height, cm	−0.38	0.04	−0.23	0.23
METABOLIC				
Fasting glucose at high-RS intervention, mg/dL	0.38	0.04	0.21	0.26
DIET				
Insoluble fiber, g	−0.20	0.28	−0.37	0.04
Kcals from fat, %	−0.13	0.49	0.39	0.03
Kcals from protein, %	−0.20	0.30	0.50	0.005
MICROBIOME ^†^ (relative abundance)				
Actinobacteria (phyla)	−0.16	0.67	−0.40	0.04
*Faecalibacterium*	−0.44	0.02	0.03	0.87

Abbreviations: kcal, kilocalories. * Note: Variables are included in the table if statistically significance correlations were found with either the low- or high-RS iAUCs. No correlative variable showed significant relationships with both potato types. ^†^ All microbiome correlations became nonsignificant when the Bonferroni correction factor was applied.

**Table 4:** Unadjusted and adjusted coefficients from linear regression that predict postprandial glucose response.

	Univariate		Multivariate	
	β Coef. (95% CI)	*p*-Value	β Coef. (95% CI)	*p*-Value
Low-RS (vs. high-RS) potato	547.65 (153.72, 941.58)	0.01	547.65 (131.61, 963.68)	0.01
*Faecalibacterium*	−69.37 (−124.15, −14.58)	0.02	−73.49 (−128.51, −18.47)	0.01
*Bacteroides*	11.26 (−11.78, 34.31)	0.33	8.69 (−14.33, 31.72)	0.45
Body mass index (kg/m^2^)	49.05 (−77.58, 175.68)	0.39	40.66 (−54.21, 135.54)	0.39
Alpha Diversity, Simpson	−5599.38 (−15,827.10, 4628.34)	0.27	110.87 (−10,209.57, 10,431.30)	0.98
Insoluble fiber, g	−50.10 (−101.24, 1.05)	0.06	−49.35 (−116.56, 17.86)	0.14
*Parabacteroides*	−70.90 (−173.86, 32.06)	0.17	−42.08 (−136.35, 52.18)	0.37
Intercept	--	--	292.52 (−9705.98, 10,291.01)	0.95

## Data Availability

Data used in the study may be available upon request to the corresponding officer.

## Supplementary Materials

The following supporting information can be downloaded at: https://www.mdpi.com/article/10.3390/nu14020268/s1, Table S1: title, Input Variables by Data Category.

## References

[1] Maziarz M.P., Preisendanz S., Juma S., Imrhan V., Prasad C., Vijayagopal P. (2017). Resistant starch lowers postprandial glucose and leptin in overweight adults consuming a moderate-to-high-fat diet: A randomized-controlled trial. Nutr. J..

[2] Nilsson A., Johansson E., Ekström L., Björck I. (2013). Effects of a brown beans evening meal on metabolic risk markers and appetite regulating hormones at a subsequent standardized breakfast: A randomized cross-over study. PLoS ONE.

[3] Nilsson A.C., Johansson-Boll E.V., Bjorck I.M. (2015). Increased gut hormones and insulin sensitivity index following a 3-d intervention with a barley kernel-based product: A randomised cross-over study in healthy middle-aged subjects. Br. J. Nutr..

[4] Bodinham C.L., Smith L., Thomas E.L., Bell J.D., Swann J.R., Costabile A., Russell-Jones D., Umpleby A.M., Robertson M.D. (2014). Efficacy of increased resistant starch consumption in human type 2 diabetes. Endocr. Connect..

[5] Bodinham C.L., Al-Mana N.M., Smith L., Robertson M.D. (2013). Endogenous plasma glucagon-like peptide-1 following acute dietary fibre consumption. Br. J. Nutr..

[6] Stewart M.L., Zimmer J.P. (2018). Postprandial glucose and insulin response to a high-fiber muffin top containing resistant starch type 4 in healthy adults: A double-blind, randomized, controlled trial. Nutrition.

[7] Hughes R.L., Horn W.H., Finnegan P., Newman J.W., Marco M.L., Keim N.L., Kable M.E. (2021). Resistant Starch Type 2 from Wheat Reduces Postprandial Glycemic Response with Concurrent Alterations in Gut Microbiota Composition. Nutrients.

[8] Robertson T.M., Alzaabi A.Z., Robertson M.D., Fielding B.A. (2018). Starchy Carbohydrates in a Healthy Diet: The Role of the Humble Potato. Nutrients.

[9] Maki K.C., Pelkman C.L., Finocchiaro E.T., Kelley K.M., Lawless A.L., Schild A.L., Rains T.M. (2012). Resistant starch from high-amylose maize increases insulin sensitivity in overweight and obese men. J. Nutr..

[10] Raatz S.K., Idso L., Johnson L.K., Jackson M.I., Combs G.F. (2016). Resistant starch analysis of commonly consumed potatoes: Content varies by cooking method and service temperature but not by variety. Food Chem..

[11] Patel H., Royall P.G., Gaisford S., Williams G.R., Edwards C.H., Warren F.J., Flanagan B.M., Ellis P.R., Butterworth P.J. (2017). Structural and enzyme kinetic studies of retrograded starch: Inhibition of α-amylase and consequences for intestinal digestion of starch. Carbohydr. Polym..

[12] Miketinas D.C., Shankar K., Maiya M., Patterson M.A. (2020). Usual dietary intake of resistant starch in US adults from NHANES 2015–2016. J. Nutr..

[13] Patterson M.A., Fong J.N., Maiya M., Kung S., Sarkissian A., Nashef N., Wang W. (2019). Chilled Potatoes Decrease Postprandial Glucose, Insulin, and Glucose-dependent Insulinotropic Peptide Compared to Boiled Potatoes in Females with Elevated Fasting Glucose and Insulin. Nutrients.

[14] Gaesser G.A., Rodriguez J., Patrie J.T., Whisner C.M., Angadi S.S. (2019). Effects of Glycemic Index and Cereal Fiber on Postprandial Endothelial Function, Glycemia, and Insulinemia in Healthy Adults. Nutrients.

[15] Parada J., Aguilera J.M. (2009). In vitro Digestibility and Glycemic Response of Potato Starch is Related to Granule Size and Degree of Gelatinization. J. Food Sci..

[16] Rajabi S., Mazloom Z., Zamani A., Tabatabaee H.R. (2015). Effect of Low Glycemic Index Diet Versus Metformin on Metabolic Syndrome. Int. J. Endocrinol. Metab..

[17] Askari M., Dehghani A., Abshirini M., Raeisi T., Alizadeh S. (2021). Glycemic index, but not glycemic load, is associated with an increased risk of metabolic syndrome: Meta-analysis of observational studies. Int. J. Clin. Pract..

[18] Livesey G., Taylor R., Livesey H.F., Buyken A.E., Jenkins D.J.A., Augustin L.S.A., Sievenpiper J.L., Barclay A.W., Liu S., Wolever T.M.S. (2019). Dietary Glycemic Index and Load and the Risk of Type 2 Diabetes: Assessment of Causal Relations. Nutrients.

[19] Jenkins D.J., Kendall C.W., Augustin L.S., Mitchell S., Sahye-Pudaruth S., Blanco Mejia S., Chiavaroli L., Mirrahimi A., Ireland C., Bashyam B. (2012). Effect of legumes as part of a low glycemic index diet on glycemic control and cardiovascular risk factors in type 2 diabetes mellitus: A randomized controlled trial. Arch. Intern. Med..

[20] Ma X.Y., Liu J.P., Song Z.Y. (2012). Glycemic load, glycemic index and risk of cardiovascular diseases: Meta-analyses of prospective studies. Atherosclerosis.

[21] Sacks F.M., Carey V.J., Anderson C.A., Miller E.R., Copeland T., Charleston J., Harshfield B.J., Laranjo N., McCarron P., Swain J. (2014). Effects of high vs low glycemic index of dietary carbohydrate on cardiovascular disease risk factors and insulin sensitivity: The OmniCarb randomized clinical trial. JAMA.

[22] Ze X., Duncan S.H., Louis P., Flint H.J. (2012). Ruminococcus bromii is a keystone species for the degradation of resistant starch in the human colon. ISME J..

[23] Schwiertz A., Lehmann U., Jacobasch G., Blaut M. (2002). Influence of resistant starch on the SCFA production and cell counts of butyrate-producing Eubacterium spp. in the human intestine. J. Appl. Microbiol..

[24] Tolhurst G., Heffron H., Lam Y.S., Parker H.E., Habib A.M., Diakogiannaki E., Cameron J., Grosse J., Reimann F., Gribble F.M. (2012). Short-chain fatty acids stimulate glucagon-like peptide-1 secretion via the G-protein-coupled receptor FFAR2. Diabetes.

[25] MacNeil S., Rebry R.M., Tetlow I.J., Emes M.J., McKeown B., Graham T.E. (2013). Resistant starch intake at breakfast affects postprandial responses in type 2 diabetics and enhances the glucose-dependent insulinotropic polypeptide—Insulin relationship following a second meal. Appl. Physiol. Nutr. Metab..

[26] Psichas A., Sleeth M.L., Murphy K.G., Brooks L., Bewick G.A., Hanyaloglu A.C., Ghatei M.A., Bloom S.R., Frost G. (2015). The short chain fatty acid propionate stimulates GLP-1 and PYY secretion via free fatty acid receptor 2 in rodents. Int. J. Obes..

[27] Korem T., Zeevi D., Zmora N., Weissbrod O., Bar N., Lotan-Pompan M., Avnit-Sagi T., Kosower N., Malka G., Rein M. (2017). Bread Affects Clinical Parameters and Induces Gut Microbiome-Associated Personal Glycemic Responses. Cell Metab..

[28] Zeevi D., Korem T., Zmora N., Israeli D., Rothschild D., Weinberger A., Ben-Yacov O., Lador D., Avnit-Sagi T., Lotan-Pompan M. (2015). Personalized nutrition by prediction of glycemic responses. Cell.

[29] (2019). Resistant Starch Assay Procedure.

[30] Wolever T.M., Jenkins D.J., Jenkins A.L., Josse R.G. (1991). The glycemic index: Methodology and clinical implications. Am. J. Clin. Nutr..

[31] Brouns F., Bjorck I., Frayn K.N., Gibbs A.L., Lang V., Slama G., Wolever T.M. (2005). Glycaemic index methodology. Nutr. Res. Rev..

[32] Banna J.C., McCrory M.A., Fialkowski M.K., Boushey C. (2017). Examining plausibility of self-reported energy intake data: Considerations for method selection. Front. Nutr..

[33] Thijs S., Op De Beeck M., Beckers B., Truyens S., Stevens V., Van Hamme J.D., Weyens N., Vangronsveld J. (2017). Comparative evaluation of four bacteria-specific primer pairs for 16S rRNA gene surveys. Front. Microbiol..

[34] Dziura J.D., Post L.A., Zhao Q., Fu Z., Peduzzi P. (2013). Strategies for dealing with missing data in clinical trials: From design to analysis. Yale J. Biol. Med..

[35] Sirimongkolkasem T., Drikvandi R. (2019). On regularisation methods for analysis of high dimensional data. Ann. Data Sci..

[36] Schreiber-Gregory D., Jackson H.M. Regulation Techniques for Multicollinearity: Lasso, Ridge, and Elastic Net. Proceedings of the SAS Conference Proceedings: Western Users of SAS Software.

[37] Organization, FaA. http://www.fao.org/3/W8079E/w8079e0a.htm.

[38] Sharma M., Li Y., Stoll M.L., Tollefsbol T.O. (2020). The Epigenetic Connection Between the Gut Microbiome in Obesity and Diabetes. Front. Genet..

[39] (2020). Dietary Guidelines for Americans, 2020–2025.

[40] https://www.arb-silva.de/.

[41] Mendes-Soares H., Raveh-Sadka T., Azulay S., Ben-Shlomo Y., Cohen Y., Ofek T., Stevens J., Bachrach D., Kashyap P., Segal L. (2019). Model of personalized postprandial glycemic response to food developed for an Israeli cohort predicts responses in Midwestern American individuals. Am. J. Clin. Nutr..

[42] Søndertoft N.B., Vogt J.K., Arumugam M., Kristensen M., Gøbel R.J., Fan Y., Lyu L., Bahl M.I., Eriksen C., Ängquist L. (2020). The intestinal microbiome is a co-determinant of the postprandial plasma glucose response. PLoS ONE.

[43] Stewart M.L., Wilcox M.L., Bell M., Buggia M.A., Maki K.C. (2018). Type-4 resistant starch in substitution for available carbohydrate reduces postprandial glycemic response and hunger in acute, randomized, double-blind, controlled study. Nutrients.

[44] Rosas-Pérez A.M., Honma K., Goda T. (2020). Sustained effects of resistant starch on the expression of genes related to carbohydrate digestion/absorption in the small intestine. Int. J. Food Sci. Nutr..

[45] Zhang X., Shen D., Fang Z., Jie Z., Qiu X., Zhang C., Chen Y., Ji L. (2013). Human gut microbiota changes reveal the progression of glucose intolerance. PLoS ONE.

[46] Xu J., Lian F., Zhao L., Zhao Y., Chen X., Zhang X., Guo Y., Zhang C., Zhou Q., Xue Z. (2015). Structural modulation of gut microbiota during alleviation of type 2 diabetes with a Chinese herbal formula. ISME J..

[47] Gurung M., Li Z., You H., Rodrigues R., Jump D.B., Morgun A., Shulzhenko N. (2020). Role of gut microbiota in type 2 diabetes pathophysiology. EBioMedicine.

