# Supplementary material for: Precision Nutrition Model Predicts Glucose Control of Overweight Females Following the Consumption of Potatoes High in Resistant Starch
Source: Nutrients. 2022 Jan 9;14(2):268. doi: 10.3390/nu14020268 (PMC8779142; doi:10.3390/nu14020268)
Supplement: Supplementary file 1 [file nutrients-14-00268-s001.zip › nutrients-1488614-supplementary.pdf]

Supplementary Table S1: Input variables by Data Category

Variable Group	Parameter
Demographics	Age, race/ethnicity (White, Hispanic, Black, Asian), sequence of potato randomization
Anthropometrics	Body mass index, waist circumference, percent body fat, percent fat free mass
Diet	Energy, fat (and % of calories), carbohydrates (and % of calories), protein (and % of calories), saturated fatty acids, monounsaturated fatty acids, polyunsaturated fatty acids, trans fatty acids, total sugar, added sugar, aspartame, available carbohydrates, total fiber, insoluble fiber, soluble fiber, glycemic index, glycemic load
α-Diversity	Shannon Index, Simpson Index
Taxa*	Proteobacteria (Phyla), Actinobacteria (Phyla), Verrucomicrobia (Phyla), Lachnospiraceae (Family), *Bacteroides, Faecalibacterium Prevotella 9, Bifidobacterium, Alistipes, Subdoligranulum, Blautia, Parabacteroides, Roseburia, Eubacterium copro group, Ruminococcus 1, Ruminococcus 2, Lachnoclostridium, Akkermansia, Ruminiclostridum, Prevotella, Eubacterium eligens (species)*

*Genus unless otherwise labeled
